# Cardiovascular Hazards of Abacavir- Versus Tenofovir-Containing Antiretroviral Therapies: Insights From an Analysis of the REPRIEVE Trial Cohort

**DOI:** 10.1093/ofid/ofaf177

**Published:** 2025-03-21

**Authors:** Emma Davies Smith, Carlos Malvestutto, Heather J Ribaudo, Carl J Fichtenbaum, Judith A Aberg, Maya Watanabe, Gerald S Bloomfield, Judith S Currier, Sarah M Chu, Kathleen V Fitch, Marissa R Diggs, Roger Bedimo, Javier Valencia, Cristina Gomez-Ayerbe, Indira Brar, Jose Valdez Madruga, Michael T Lu, Pamela S Douglas, Markella V Zanni, Steven K Grinspoon

**Affiliations:** Center for Biostatistics in AIDS Research, Harvard T. H. Chan School of Public Health, Boston, Massachusetts, USA; Division of Infectious Diseases, Ohio State University Medical Center, Columbus, Ohio, USA; Center for Biostatistics in AIDS Research, Harvard T. H. Chan School of Public Health, Boston, Massachusetts, USA; Division of Infectious Diseases, University of Cincinnati College of Medicine, Cincinnati, Ohio, USA; Division of Infectious Diseases, Icahn School of Medicine at Mount Sinai, New York, New York, USA; Center for Biostatistics in AIDS Research, Harvard T. H. Chan School of Public Health, Boston, Massachusetts, USA; Department of Medicine, Duke Global Health Institute and Duke Clinical Research Institute, Duke University, Durham, North Carolina, USA; Division of Infectious Diseases, David Geffen School of Medicine, University of California, Los Angeles, Los Angeles, California, USA; Metabolism Unit, Massachusetts General Hospital and Harvard Medical School, Boston, Massachusetts, USA; Metabolism Unit, Massachusetts General Hospital and Harvard Medical School, Boston, Massachusetts, USA; Metabolism Unit, Massachusetts General Hospital and Harvard Medical School, Boston, Massachusetts, USA; Department of Medicine, UT Southwestern, Dallas, Texas, USA; San Miguel Clinical Research Site, Asociacion Civil Impacta Salud y Educación, Lima, Peru; Unit of Infectious Diseases, Hospital Universitario Virgen de la Victoria, Instituto de Investigación Biomédica de Málaga y Plataforma en Nanomedicina, Málaga, Spain; Division of Infectious Disease, Henry Ford Hospital, Detroit, Michigan, USA; Centro de Referencia e Treinamento DST/AIDS, São Paulo, Brazil; Cardiovascular Imaging Research Center, Department of Radiology, Massachusetts General Hospital and Harvard Medical School, Boston, Massachusetts, USA; Duke Clinical Research Institute, Duke University School of Medicine, Durham, North Carolina, USA; Metabolism Unit, Massachusetts General Hospital and Harvard Medical School, Boston, Massachusetts, USA; Metabolism Unit, Massachusetts General Hospital and Harvard Medical School, Boston, Massachusetts, USA

**Keywords:** abacavir, antiretroviral therapy, major adverse cardiovascular events, REPRIEVE, tenofovir

## Abstract

**Background:**

Prior analyses suggest that the nucleoside reverse transcriptase inhibitor (NRTI) abacavir (ABC), but not tenofovir (TFV), is associated with a 2-fold increase in the hazard of myocardial infarction. the Randomized Trial to Prevent Vascular Events in HIV (REPRIEVE) is ideally suited to evaluate the role of ABC and the TFV backbones, tenofovir alafenamide (TAF) and tenofovir disoproxil fumarate (TDF), in major adverse cardiovascular events (MACE).

**Methods:**

We compared hazard of first MACE among people living with human immunodeficiency virus (HIV) at low-to-moderate cardiovascular risk using ABC (n = 883), TAF (n = 957), and TDF (n = 4274) at entry. Overlap weights balanced biasing factors, including age, sex at birth, atherosclerotic cardiovascular disease risk, CD4 count, estimated glomerular filtration rate, and anchor antiretroviral therapy. Associations between entry NRTI and MACEs were estimated using a marginal Cox proportional hazards model. Change of NRTI, or “switching,” was common during follow-up. Additional associations were estimated by further censoring at first switch and applying time-updated inverse probability of censoring weighting (IPCW).

**Results:**

Baseline-adjusted associations suggest clinically relevant increases in hazard of first MACE for ABC versus TAF (hazard ratio [HR], 1.5 [95% confidence interval {CI}, .9–2.3]) and ABC versus TDF (HR, 1.4 [95% CI, .9–2.1]), but not TAF versus TDF (HR, 0.9 [95% CI, .6–1.5]). With censoring at switch, HRs increased to 1.6 (95% CI, .9–2.7) for ABC versus TAF, 2.0 (95% CI, 1.2–3.4) for ABC versus TDF, and 1.2 (95% CI, .7–2.2) for TAF versus TDF. The largest HR observed was for ABC versus TDF and myocardial infarction (IPCW HR, 3.5 [95% CI, 1.3–9.4]).

**Conclusions:**

Antiretroviral therapies with ABC backbones are associated with an increase in MACE compared to TFV backbones among people living with HIV at low-to-moderate cardiovascular risk.

**Clinical Trials Registration:**

NCT02344290.

## Teaser

Time-updated analyses of the REPRIEVE cohort suggest that antiretroviral therapies featuring abacavir backbones increase hazard of major adverse cardiovascular events by at least 40% compared to tenofovir backbones, with little difference between tenofovir alafenamide and tenofovir disoproxil fumarate.

The risk of cardiovascular disease (CVD) in people living with human immunodeficiency virus (HIV) is up to twice that of the general population. Traditional risk factors, including cigarette smoking and diabetes mellitus type 2 [1], and some nontraditional risk factors, such as hepatitis C coinfection [2], are more common among people living with HIV (PWH). Even after controlling for these risk factors, excess risk persists that is driven, in part, by chronic inflammation and residual immune activation [3, 4]. Untreated HIV also contributes to increased incidence of CVD events compared to HIV that is virally suppressed [5]. While antiretroviral therapy (ART) can maintain viral control indefinitely, some ART agents have been reported to increase the incidence of major adverse cardiovascular events (MACE) in PWH.

In 2007, the Data Collection on Adverse Events of Anti-HIV Drugs (D:A:D) study, a prospective observational cohort study of 23 490 participants, reported that the cumulative exposure of some protease inhibitors (ie, lopinavir [LPV]–ritonavir [RTV]) was associated with increased risk of myocardial infarction (MI) [6]. In a subsequent analysis investigating the impact of nucleoside reverse transcriptase inhibitors (NRTIs) that included 33 347 participants with 517 reported MI events, recent (within 6 months of the MI event) but not cumulative or past use of abacavir (ABC) was associated with an increased risk of MI (risk ratio 1.90 [95% confidence interval {CI}, 1.47–2.45]; *P* = .001) [7]. Multiple cohort studies and meta-analyses have since corroborated this association with recent exposure to ABC [8–10] and with cumulative use of ABC [11, 12]. However, other studies have not shown a similar association [13–16].

Critics of analyses reporting increased risk of CVD associated with ABC raised concerns about possible channeling bias related to avoidance of the alternative NRTI tenofovir disoproxil fumarate (TDF) in favor of ABC for patients with chronic kidney disease (CKD), a known risk factor for CVD [9, 17–19], as well as the possible contribution of older, more toxic, antiretroviral agents that are no longer regularly used. A recent analysis by the International Cohort Consortium of Infectious Disease (RESPOND) Study Group, a multinational cohort of 29 340 PWH from European and Australian populations followed between 2012 and 2019 with contemporary ART use, corroborated an increased CVD risk associated with recent ABC use (incidence rate ratio 1.40 [95% CI, 1.20–1.64]). As in earlier reports, association was not observed with past ABC use. The association did not differ according to CVD or CKD risk strata [20].

Reports of the association between NRTI use and CVD risk from global cohorts that include PWH from low- and middle-income countries using contemporary ART options are lacking. In this analysis, we compare the hazards of ART regimens featuring abacavir backbones versus tenofovir backbones with respect to MACE within the Randomized Trial to Prevent Vascular Events in HIV (REPRIEVE), a global cardiovascular-focused trial of PWH with prospective assessment of key cardiovascular risk factors, and prespecified adjudication of MACE and its subcomponents.

## METHODS

### Participants

REPRIEVE investigated whether statin therapy is protective for MACE among PWH at low-to-moderate cardiovascular risk. Between March 2015 and July 2019, 7769 PWH were randomly allocated according to a 1:1 ratio to receive pitavastatin or placebo [21]. Sites were located in 5 global burden of disease (GBD) regions spanning 12 countries: high-income countries (Canada, United States excluding Puerto Rico, Spain); Latin America and Caribbean (Brazil, Haiti, Peru, Puerto Rico); sub-Saharan Africa (Botswana, South Africa, Uganda, Zimbabwe); Southeast Asia (Thailand); and South Asia (India). The trial was stopped early in March 2023 due to efficacy, with final results suggesting a 36% reduction in the incidence of first MACE among those randomized to pitavastatin versus placebo [22, 23].

Relevant inclusion criteria include diagnosis of HIV; low-to-moderate risk of atherosclerotic cardiovascular disease (ASCVD); receipt of stable ART; CD4^+^ count >100 cells/μL; and satisfaction of laboratory thresholds including estimated glomerular filtration rate (eGFR) ≥60 mL/minute/1.73 m^2^. Relevant exclusion criteria include the previous occurrence of ASCVD-related events [23].

Following randomization (“entry”), participants returned every 4 months for a study visit and every 1 month for a safety visit, and targeted physical examinations and laboratory testing were conducted every 12 months. Final study visits were conducted between April and August 2023.

### Exposures

ART regimen details were collected at entry into REPRIEVE, including start date of current regimen prior to REPRIEVE entry. Details and timings of any ART changes were documented at study visits. For the present analysis, we define 3 exposure groups based on recorded NRTI use at entry: (1) tenofovir disoproxil fumarate, without abacavir (“TDF”); (2) tenofovir alafenamide, without abacavir (“TAF”); and (3) abacavir, with or without TAF or TDF (“ABC”).

To compose a fully effective ART regimen, NRTIs are commonly combined with an “anchor drug.” Three anchor drug classes at entry are included: (1) integrase strand transfer inhibitor (INSTI); (2) nonnucleoside reverse transcriptase inhibitor (NNRTI); or (3) protease inhibitor (PI).

REPRIEVE participants were ineligible for inclusion within our analysis if they could not be categorized within the defined NRTI or anchor drug classes, including participants taking anchor drug combinations (eg, INSTI with an NNRTI).

### Endpoints

We consider the same primary endpoint as REPRIEVE: the time to first MACE [23]. MACE is a composite of cardiovascular death (including deaths from undetermined cause), MI, hospitalization for unstable angina, stroke, transient ischemic attack, peripheral arterial ischemia, or revascularization. As secondary endpoints, we consider time to first hard MACE and its components: cardiovascular death, stroke, and MI.

All MACE events were independently adjudicated by members of the Thrombolysis in Myocardial Infarction (TIMI) team blinded to statin assignment [23].

### Associations

Crossover, or “switching,” between exposure groups during follow-up can dilute or bias associations. Changes to ART generally, or NRTI specifically, are expected and common among PWH for multiple reasons including concern for adverse effects, possible drug–drug interactions with concomitant medications, or patient choice for more convenient regimens.

To robustly differentiate the hazards of our primary exposures, we estimated 2 sets of associations. First, hazard ratios (HRs) comparing NRTI at entry, ignoring switches during follow-up; in a randomized controlled trial (RCT), this association would correspond to the intention-to-treat effect. Second, HRs comparing NRTI at entry, with additional censoring at first switch. Inverse probability of censoring weighting (IPCW) is then used to upweight individuals remaining on their NRTI who best represent those censored [24]. In an RCT, this association would correspond to the hypothetical effect if it were possible to prevent all switches [25].

Unadjusted and baseline-adjusted associations, without and with censoring at switch and IPCW, are reported for all endpoints.

### Adjustment for Confounding, Selection, and Precision Factors

Co-authors (C. M., C. J. F., J. A. A., and S. K. G.) identified factors likely to influence both NRTI selection and MACE occurrence. Adjustment factors defined at entry are randomized intervention (pitavastatin or placebo), age, sex at birth, randomized arm, anchor drug class, ASCVD risk score, substance use, smoking status, nadir CD4 cell count, and lifetime duration of ART. Time-updated adjustment factors are body mass index (BMI), incident or preexisting hypertension, incident or preexisting diabetes, high-density lipoprotein (HDL), eGFR, and CD4 cell count. BMI, HDL, and CD4 cell count were captured at annual clinic visits. Incident hypertension, incident diabetes, and changes in eGFR were captured as adverse events. The visit schedule and measurement details can be found in the REPRIEVE protocol [23].

### Censoring

Loss to follow-up and competing noncardiovascular deaths are right-censored for all associations. As in REPRIEVE, we assume loss to follow-up is independent of MACE, or “noninformative.” Hypothetical associations further censor participants at the time of their first NRTI switch [24]. Censoring at switch is assumed to be informative, but reasons for switching were not captured. We assume that the same factors influencing NRTI also influence first NRTI switch.

### Missing Data

A complete case analysis is performed by excluding the small proportion of participants with incomplete adjustment factors at entry. Last observation carried forward is used to impute time-updated adjustment factors.

### Statistical Methods

Hazard ratios are estimated using marginal structural Cox proportional hazards models with NRTI at entry as the sole covariate. Generalized overlap weights are used for baseline adjustment, and estimated by a multinomial logistic model that regresses NRTI on adjustment factors at entry [26]. Use of overlap weights emphasizes participants with equal likelihood of receiving ABC, TAF, or TDF at entry into REPRIEVE, or the “equipoise” population.

With censoring at switch, HRs are approximated using a pooled logistic model with polynomial splines for follow-up month [27]. Time-updated weights are obtained by cumulatively multiplying baseline overlap weights by monthly, stabilized IPCWs [28]. IPCWs are estimated separately for each NRTI using a pooled logistic model for first switch, on time-updated covariates, with splines for month.

Robust 95% confidence intervals (CIs) are reported for all associations. No adjustment is made for multiple comparisons. All analyses were performed using SAS version 9.4 software.

### Sensitivity Analyses

TAF and ABC was almost exclusively used in high-income countries, likely due to availability of TAF-containing single-tablet ART combinations, precluding GBD as an adjustment factor due to positivity violations [29]. For similar reasons, duration of use prior to entry was also shorter for TAF than TDF and ABC. Race was also identified as an important adjustment factor, but variation in race was minimal outside of high-income countries in REPRIEVE. Sensitivity analyses estimate baseline-adjusted associations among (1) participants in high-income countries; (2) participants in high-income countries, with additional adjustment for race (Black vs non-Black); and (3) participants using their entry NRTI for <1 year.

Censoring at switch with IPCW was not performed for either sensitivity analysis, as events and sample sizes within subgroups were already limited.

## RESULTS

### Participants

Of the 7769 PWH randomized by REPRIEVE, 6356 (82%) were eligible for this analysis. Among ineligible participants, the 4 most common ART regimens featured combinations of the NRTI zidovudine (ZDV) without ABC, TDF, or TAF: lamivudine (3TC)/ZDV/nevirapine (22%), 3TC/ZDV/efavirenz (17%), 3TC/ZDV/RTV/atazanavir (5%), and 3TC/ZDV/RTV/LPV (4%). Two hundred forty-two eligible participants were excluded from the analysis due to missing baseline adjustment factors, resulting in the inclusion of 6114 total REPRIEVE participants within our cohort. The median follow-up time was 67 (interquartile range [IQR], 57–77) months.

Baseline demographics and CVD risk factors for the 6114 included participants are summarized by entry NRTI in Table 1, and baseline HIV and ART characteristics are summarized in Table 2. In comparison to TDF users (n = 4274), ABC (n = 883) and TAF (n = 957) users were older with higher ASCVD risk scores and lower eGFRs, less likely to be female, and more likely to have a history of former substance use. Almost 70% of those on ABC and TAF used INSTIs, while 63% of those on TDF used NNRTIs. Among those on ABC, approximately 7% were recorded as also using TDF (n = 51) or TAF (n = 7).

**Table 1.** Baseline Demographics, Cardiovascular Disease Risk Factors, and Major Adverse Cardiovascular Events by Primary Exposure (Nucleoside Reverse Transcriptase Inhibitor at Entry), as Observed and Following Overlap Weighting (Baseline Adjustment)

Characteristic	Observed			Overlap Weighted		
	ABC (n = 883)	TAF (n = 957)	TDF (n = 4272)	ABC (n = 307)	TAF (n = 310)	TDF (n = 315)
Age, y						
40–49	342 (39)	360 (38)	2329 (54)	127 (42)	130 (42)	130 (41)
50–59	433 (49)	487 (51)	1667 (39)	149 (48)	150 (48)	155 (49)
≥60	108 (12)	110 (11)	278 (7)	31 (10)	30 (10)	31 (10)
Female sex at birth	202 (23)	171 (18)	1572 (37)	68 (22)	65 (21)	64 (20)
GBD region by race						
High income—Black	302 (34)	366 (38)	663 (16)	98 (32)	114 (37)	93 (30)
High income—non-Black	449 (51)	579 (61)	955 (22)	155 (50)	192 (62)	133 (42)
Latin America and Caribbean	83 (9)	5 (1)	976 (23)	35 (11)	2 (1)	44 (14)
Sub-Saharan Africa	19 (2)	0 (0)	881 (21)	7 (2)	0 (0)	24 (8)
Southeast/East Asia	22 (2)	2 (0)	501 (12)	9 (3)	1 (0)	13 (4)
South Asia	8 (1)	5 (1)	298 (7)	3 (1)	3 (1)	7 (2)
Randomized arm						
Pitavastatin (vs placebo)	450 (51)	483 (50)	2130 (50)	156 (51)	158 (51)	158 (50)
Substance use						
Current	20 (2)	29 (3)	77 (2)	8 (3)	8 (2)	9 (3)
Former	394 (45)	486 (51)	966 (23)	131 (43)	140 (45)	147 (47)
Never	469 (53)	442 (46)	3231 (76)	168 (55)	163 (53)	160 (51)
ASCVD risk score, %						
0 to <2.5	163 (18)	137 (14)	1501 (35)	58 (19)	57 (18)	59 (19)
2.5 to <5	227 (26)	224 (23)	1171 (27)	81 (27)	82 (26)	85 (27)
5 to <7.5	250 (28)	284 (30)	926 (22)	89 (29)	88 (28)	94 (30)
7.5–10	157 (18)	210 (22)	460 (11)	53 (17)	56 (18)	52 (17)
>10	86 (10)	102 (11)	216 (5)	25 (8)	28 (9)	26 (8)
BMI, kg/m^2^						
<25	340 (39)	295 (31)	1971 (46)	116 (38)	109 (35)	115 (36)
25–29.9	333 (38)	378 (39)	1414 (33)	112 (37)	120 (39)	118 (37)
≥30	210 (24)	284 (30)	889 (21)	79 (26)	81 (26)	82 (26)
HDL (mg/dL), <40 if male or <50 if female	243 (28)	260 (27)	1587 (37)	91 (30)	92 (30)	95 (30)
Hypertensive (yes)	345 (39)	373 (39)	1410 (33)	114 (37)	118 (38)	113 (36)
Diabetic (yes)	6 (1)	4 (0)	20 (0)	2 (1)	1 (0)	3 (1)
Smoking status						
Current	267 (30)	298 (31)	963 (23)	91 (30)	93 (30)	94 (30)
Former	260 (29)	279 (29)	967 (23)	89 (29)	90 (29)	97 (31)
Never	356 (40)	380 (40)	2344 (55)	127 (41)	127 (41)	125 (40)
eGFR (mL/min per 1.73 mm^2^)						
<60	80 (9)	62 (6)	60 (1)	16 (5)	17 (5)	17 (6)
60 to <90	472 (53)	550 (57)	1523 (36)	163 (53)	170 (55)	172 (55)
≥90	331 (37)	345 (36)	2691 (63)	127 (42)	124 (40)	126 (40)
MACE	48 (5)	36 (4)	99 (2)	16 (5)	10 (3)	13 (4)

Data are presented as No. (%). For overlap weighted, No. (%) represents the sum (proportion) of weights within each category.

Abbreviations: ABC, abacavir; ASCVD, atherosclerotic cardiovascular disease; BMI, body mass index; eGFR, estimated glomerular filtration rate; GBD, global burden of disease; HDL, high-density lipoprotein; MACE, major adverse cardiovascular event; TAF, tenofovir alafenamide; TDF, tenofovir disoproxil fumarate.

**Table 2.** Baseline Human Immunodeficiency Virus and Antiretroviral Therapy Characteristics by Primary Exposure (Nucleoside Reverse Transcriptase Inhibitor at Entry), as Observed and Following Overlap Weighting (Baseline Adjustment)

Characteristic	Observed			Overlap Weighted		
	ABC (n = 883)	TAF (n = 957)	TDF (n = 4272)	ABC (n = 307)	TAF (n = 310)	TDF (n = 315)
Anchor drug class						
NNRTI	149 (17)	181 (19)	2683 (63)	72 (24)	70 (22)	73 (23)
INSTI	583 (66)	674 (70)	616 (14)	181 (59)	189 (61)	188 (60)
PI	151 (17)	102 (11)	975 (23)	54 (18)	51 (17)	54 (17)
Duration of current NRTI, y						
<1	334 (38)	628 (66)	896 (21)	111 (36)	211 (68)	88 (28)
1–2	325 (37)	301 (31)	1332 (31)	108 (35)	90 (29)	108 (34)
≥3	224 (25)	27 (3)	2046 (48)	87 (28)	8 (3)	120 (38)
Missing	0	1	0	0	1	0
CD4 count, cells/μL						
<200	27 (3)	33 (3)	116 (3)	9 (3)	10 (3)	8 (3)
200–349	91 (10)	92 (10)	436 (10)	31 (10)	32 (10)	34 (11)
350–499	154 (17)	153 (16)	775 (18)	51 (17)	53 (17)	52 (17)
≥500	611 (69)	679 (71)	2947 (69)	216 (70)	215 (69)	221 (70)
Nadir CD4 count, cells/μL						
<50	182 (21)	183 (19)	685 (16)	59 (19)	59 (19)	60 (19)
50–199	267 (30)	252 (26)	1296 (30)	88 (29)	89 (29)	89 (28)
200–349	242 (27)	251 (26)	1245 (29)	86 (28)	83 (27)	91 (29)
≥350	192 (22)	271 (28)	1048 (25)	73 (24)	80 (26)	75 (24)
Lifetime ART duration, y						
<5	168 (19)	166 (17)	1212 (28)	60 (20)	62 (20)	61 (19)
5–9	220 (25)	286 (30)	1360 (32)	89 (29)	88 (28)	91 (29)
≥10	495 (56)	505 (53)	1702 (40)	157 (51)	160 (52)	163 (52)

Data are presented as No. (%). For overlap weighted, No. (%) represents the sum (proportion) of weights within each category.

Abbreviations: ABC, abacavir; ART, antiretroviral therapy; INSTI, integrase strand transfer inhibitor; NNRTI, nonnucleoside reverse transcriptase inhibitor; NRTI, nucleoside reverse transcriptase inhibitor; PI, protease inhibitor; TAF, tenofovir alafenamide; TDF, tenofovir disoproxil fumarate.

A total of 183 first MACE events were observed: 5% of ABC and 4% of TAF users experienced at least 1 MACE compared to 2% of TDF users. Sixty-four percent of MACEs occurred while on entry NRTI.

**Figure 1.**
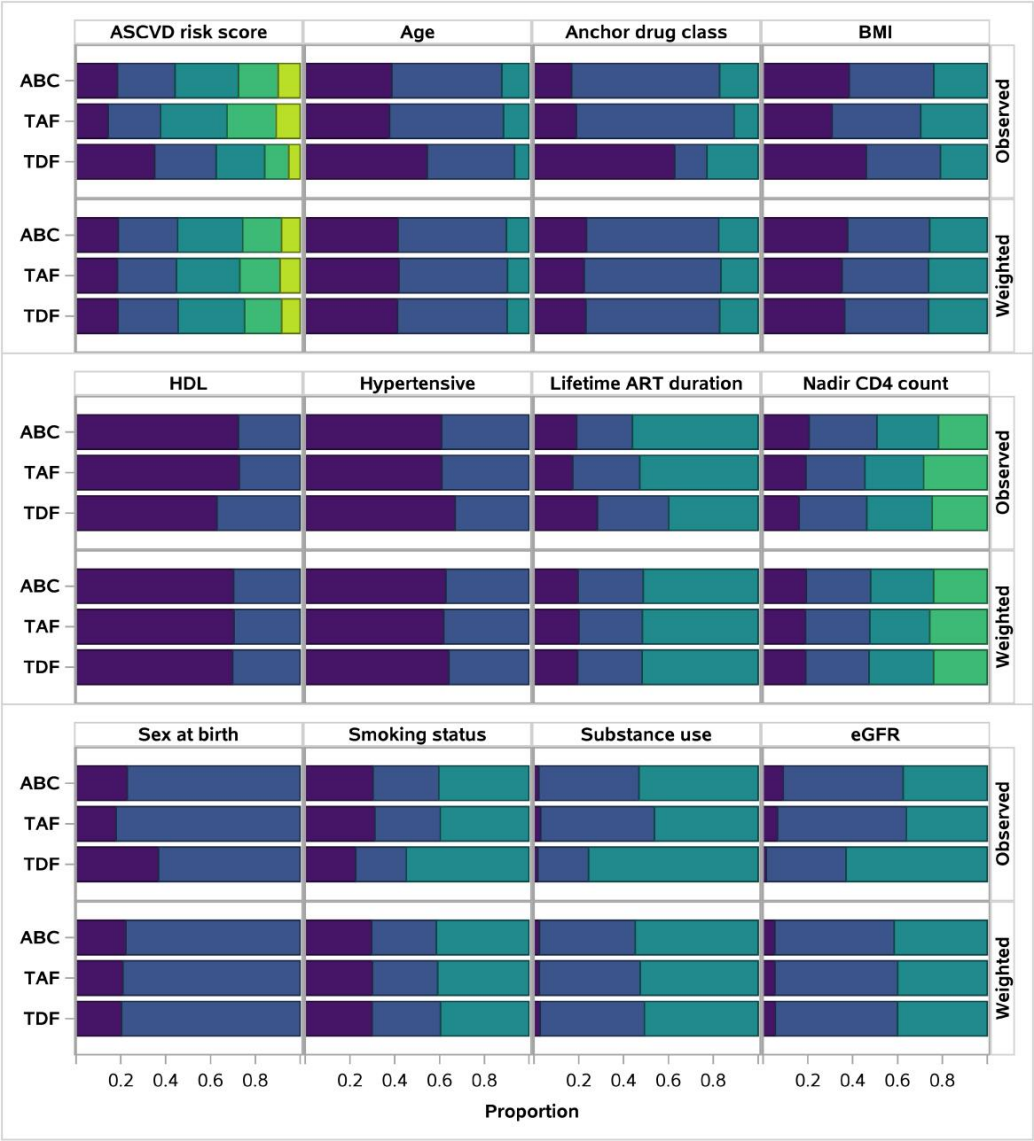
Balance in baseline adjustment factors without (top panels) and with (bottom) overlap weighting. Segments represent categories of named characteristics, in same order as Table 1. For example, observed atherosclerotic cardiovascular disease scores were lower for tenofovir disoproxil fumarate than abacavir or tenofovir alafenamide, but similar after weighting. Randomized arm and CD4 cell count omitted due to balance pre- and postweighting. Diabetes omitted due to small sample sizes. Abbreviations: ABC, abacavir; ART, antiretroviral therapy; ASCVD, atherosclerotic cardiovascular disease; BMI, body mass index; eGFR, estimated glomerular filtration rate; HDL, high-density lipoprotein; TAF, tenofovir alafenamide; TDF, tenofovir disoproxil fumarate.

Figure 1 and Table 1 summarize baseline characteristics before and after application of overlap weights (baseline adjustment), and suggest almost perfect balance between weighted NRTI groups.

### Primary Endpoints

**Figure 2.**
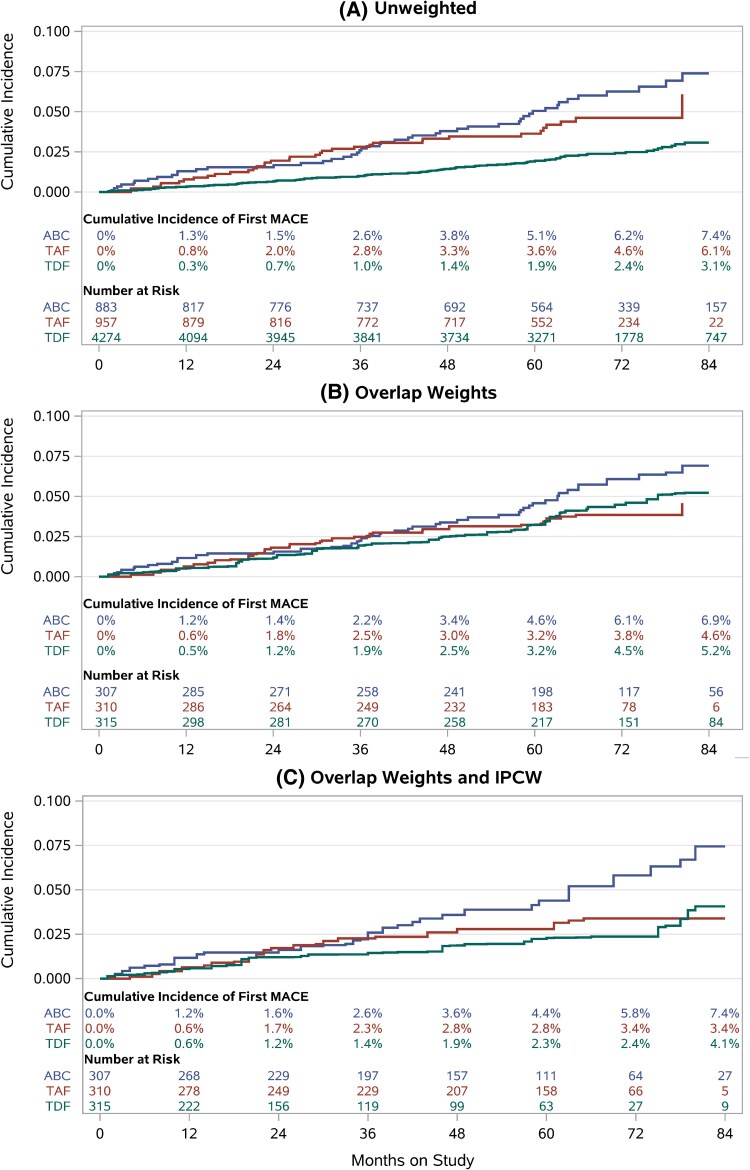
Cumulative incidence curves up to 84 months of follow-up with no adjustment (*A*), baseline adjustment (*B*), and baseline and switch adjustment (*C*). Blue curve represents abacavir; red, tenofovir alafenamide; and green, tenofovir disoproxil fumarate. Abbreviations: ABC, abacavir; IPCW, inverse probability of censoring weighting; MACE, major adverse cardiovascular event; TAF, tenofovir alafenamide; TDF, tenofovir disoproxil fumarate.

Cumulative incidence of first MACE is presented in Figure 2. Without adjustment for baseline imbalances or switches (Figure 2*A*), curves were similar for ABC and TAF but much lower for TDF. When NRTI groups were balanced per Figure 1, smaller yet visible differences remained between ABC and TDF (Figure 2*B*). The relative position of TAF was no longer obvious, with greater incidence than ABC early in follow-up and lower incidence than TDF later. After censoring at switch with IPCW (Figure 2*C*), TAF and TDF both had lower incidence than ABC. However, TAF had slightly elevated incidence compared to TDF.

Hazard ratios are reported for all endpoints in Table 3. Noting large imbalances between NRTIs at entry (Figure 1), unadjusted associations suggest that the hazard of first MACE does not differ between ABC and TAF use at entry, and both have greater hazards than TDF use at entry.

**Table 3.** Estimated Pairwise Hazard Ratios and Corresponding 95% Confidence Intervals for the Primary Endpoint of Major Adverse Cardiovascular Events (MACE) and the Secondary Endpoints of Hard MACE, Myocardial Infarction, Stroke, and Cardiovascular Death

Event (Observed)	Adjustment	ABC vs TAF	ABC vs TDF	TAF vs TDF
MACE (183)	None	1.3 (.8–2.0)	2.5 (1.8–3.6)	2.0 (1.3–2.9)
	Baseline	1.5 (.9–2.3)	1.4 (.9–2.1)	0.9 (.6–1.5)
	Baseline + Switch	1.6 (.9–2.7)	2.0 (1.2–3.4)	1.2 (.7–2.2)
Hard MACE (121)	None	1.1 (.6–1.8)	2.5 (1.6–3.8)	2.3 (1.4–3.5)
	Baseline	1.2 (.7–2.1)	1.3 (.8–2.2)	1.1 (.6–1.9)
	Baseline + Switch	1.5 (.8–2.7)	1.8 (1.0–3.4)	1.2 (.6–2.4)
MI (57)	None	1.1 (.5–2.1)	3.7 (2.0–6.9)	3.5 (1.9–6.6)
	Baseline	1.4 (.7–2.8)	1.9 (.9–4.2)	1.4 (.6–3.1)
	Baseline + Switch	1.4 (.6–3.0)	3.5 (1.3–9.4)	2.5 (.9–7.0)
Stroke (50)	None	1.5 (.6–3.6)	2.1 (1.1–4.1)	1.4 (.7–3.1)
	Baseline	1.5 (.6–3.8)	1.1 (.5–2.5)	0.8 (.3–1.9)
	Baseline + Switch	2.9 (.9–9.8)	1.3 (.5–3.4)	0.5 (.1–1.5)
CV death (21)	None	0.9 (.2–3.6)	1.6 (.5–5.0)	1.8 (.6–5.5)
	Baseline	0.9 (.1–3.7)	1.1 (.3–3.8)	1.3 (.4–4.5)
	Baseline + Switch	0.9 (.2–4.4)	1.8 (.4–7.6)	1.9 (.4–8.7)

Data are presented as hazard ratios (95% confidence intervals). Three adjustment strategies are presented: no adjustment, baseline only (overlap weighting), and baseline + switch (overlap weighting + inverse probability of censoring weighting).

Abbreviations: ABC, abacavir; CV, cardiovascular; MACE, major adverse cardiovascular event; MI, myocardial infarction; TAF, tenofovir alafenamide; TDF, tenofovir disoproxil fumarate.

Baseline-adjusted estimates suggest that ABC is associated with a 40% increase in the hazard of first MACE when compared to TDF (HR, 1.4 [95% CI, .9–2.1]) and 50% increase when compared to TAF (HR, 1.5 [95% CI, .9–2.3]). While statistically nonsignificant, CIs suggest the data are predominantly compatible with relative harm of ABC. There appeared to be little difference between TAF and TDF (HR, 0.9 [95% CI, .6–1.5]).

**Figure 3.**
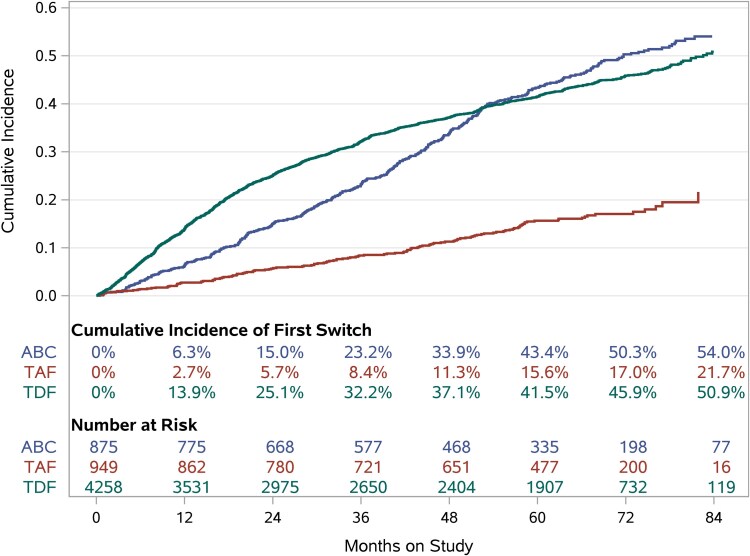
Cumulative incidence of first switch during follow-up by nucleoside reverse transcriptase inhibitor. Blue curve represents abacavir (ABC); red, tenofovir alafenamide (TAF); and green, tenofovir disoproxil fumarate (TDF).

The number of NRTI switches per participant ranged from 0 to 7: 62% of participants never switched their NRTI, 29% switched once, 6% twice, and 1.5% thrice. Figure 3 presents the cumulative incidence of first switch over follow-up by NRTI. Overall, the median time to first switch was 22 (IQR, 10–42) months among those who switched. Table 4 summarizes changes to NRTI at first switch: 78.0% and 16.3% stopped TDF and ABC, respectively, while 58.5% started TAF.

**Table 4.** Nucleoside Reverse Transcriptase Inhibitor (NRTI) Strategy at Entry (Row) and at First Switch (Column) Among 2309 Participants Who Switched Their NRTI Strategy at Least Once

NRTI at Entry	NRTI at First Switch					Total
	ABC	TAF	TDF	Other	No ART	
ABC	…	175 46.5% 13.0%	40 10.6% 67.8%	125 33.2% 23.7%	36 9.6% 22.9%	376 16.3%
TAF	10 7.5% 4.6%	…	19 14.3% 32.2%	80 60.2% 15.2%	24 18.1% 15.3%	133 5.8%
TDF	206 11.4% 95.4%	1175 65.3% 87.0%	…	322 17.9% 61.1%	97 5.4% 61.8%	1800 78.0%
Total	216 9.4%	1350 58.5%	59 2.6%	527 22.8%	157 6.8%	2309 100%

Data are presented as No, with row % and column % below. “Other” indicates ART without ABC, TAF, or TDF. Diagonal corresponds to no switch.

Abbreviations: ABC, abacavir; ART, antiretroviral therapy; NRTI, nucleoside reverse transcriptase inhibitor; TAF, tenofovir alafenamide; TDF, tenofovir disoproxil fumarate.

With censoring at first switch and IPCW, the HR comparing ABC to TDF increased to 2.0 (95% CI, 1.2–3.4), while TAF versus TDF increased to 1.2 (95% CI, .7–2.2). A minor increase in the HR comparing ABC to TAF to 1.6 (95% CI, .9–2.7) was also observed.

### Secondary Endpoints

Adjusted associations comparing ABC to TAF or TDF were generally greater than 1 for hard MACE and its components (Table 3). HRs comparing ABC to TDF were also generally greater than those for TAF, except for stroke. The largest HR estimated was that of MI for ABC versus TDF (IPCW HR, 3.5 [95% CI, 1.3–9.4]), and the second largest was that of stroke for ABC versus TAF (IPCW HR, 2.9 [95% CI, .9–9.8]).

### Sensitivity Analyses

The majority of ABC (n = 751 [84%]) and TAF (n = 945 [98%]) users but a minority of TDF (n = 1618 [39%]) users lived in high-income GBD regions at entry. Sensitivity analyses limited to high-income GBD regions showed similar elevations in MACE hazard for ABC compared to TAF (HR, 1.6 [95% CI, 1.0–2.5]) and TDF (HR, 1.3 [95% CI, .8–2.1]) with little difference between TAF and TDF (HR, 0.8 [95% CI, .5–1.4]). Results did not differ with additional adjustment for race.

The duration of TAF use immediately prior to entry (median, 8 [IQR, 3–14] months) was much shorter than that of ABC (median, 18 [IQR, 7–39] months) and TDF (median, 35 [IQR, 15–64] months). Sensitivity analyses limited to <1 year of current NRTI use at entry (ABC, n = 334; TAF, n = 628; TDF, n = 896) also demonstrated elevated MACE hazard for ABC compared to TAF (HR, 1.5 [95% CI, .7–3.2]) and TDF (HR, 1.5 [95% CI, .7–3.5]), and no difference between TAF and TDF (HR, 1.0 [95% CI, .5–2.1]).

## DISCUSSION

Our retrospective analysis of the REPRIEVE cohort suggests that ART regimens with ABC backbones are generally associated with an increased hazard of first MACE and its components when compared with the TFV backbones, TAF and TDF, after adjusting for imbalances between NRTI characteristics at entry. Similar associations were observed when restricted to participants in high-income countries and NRTI initiation within 1 year of entry, and larger effect sizes were observed when censoring at first NRTI switch with IPCW was applied. Secondary analyses also suggest a large increase in the hazard of ABC compared to TDF with respect to MI. Associations align with previous findings from the D:A:D [7] and RESPOND [20] cohort studies and a systematic review of 17 RCTs and case-control studies [10].

The hazards of alternative tenofovir backbones, TAF and TDF, appeared similar across MACE and hard MACE. Interestingly, the relative hazards of TAF versus TDF appeared to vary by component, with TAF having greater hazard of MI and cardiovascular death compared to TDF but lower hazard of stroke. However, component associations should be interpreted cautiously due to few events. Relative differences may be driven by the inherent lipid-lowering and weight-suppressing properties of TDF versus TAF [30, 31]. As duration of TAF prior to entry was much shorter than that of TDF, minor differences in hazards may also reflect prevalent-user bias, disappearing for MACE when restricted to NRTI initiation within 1 year of entry.

Various putative mechanisms to explain the effect of ABC on MACE risk have been proposed. A previous RCT that measured brachial artery ultrasound for flow-mediated dilation (FMD) as a surrogate marker of endothelial dysfunction in PWH starting ABC- or TDF-containing ART regimens found no significant differences in FMD or markers of inflammation or coagulation [32]. More evidence has been gathered supporting ABC-induced platelet aggregation and hyperreactivity [33, 34], and leukocyte recruitment driving noncalcified atherosclerotic plaque formation [35]. Supporting this hypothesis, an RCT of participants on ABC-containing ART randomized to switch to TAF or continue on ABC found that switching to TAF from ABC resulted in decreased platelet reactivity to platelet agonists, suggesting that platelet dysfunction is a viable mechanism for the increased risk of MACEs associated with ABC use [36]. It is not clear, however, how this apparently reversible effect reconciles with the reports of increased cumulative risk beyond 6 months [11, 12, 37].

In this analysis, we controlled for potential channeling bias by adjusting for key cardiovascular risk factors [38], used time-updated IPCW in analyses accounting for NRTI switches [24], and performed sensitivity analyses on baseline ART duration. Nonetheless, there are some limitations. First, we do not consider distinct regimens or interactions with anchor drug class (eg, INSTIs, PIs, or NNRTIs) here due to sample size. Anchor drug class at entry is included as an adjustment factor due to its potential association with both NRTIs and MACE, but we cannot investigate its effects on MACE directly. Additional analyses may provide insight into the mechanisms through which specific ART impacts CVD risk. Second, other studies have suggested that timing of ABC exposure is relevant, particularly within the past 6 months [20, 39]. Future REPRIEVE analyses will explore the impact of any former or current ABC exposure compared to no exposure at entry on MACE risk [37], Third, we do not assess NRTI initiation as our exposure, as in an RCT. Rather, we assess primarily prevalent, or existing, NRTI use as our exposure. Consideration of prevalent exposure can result in survival bias [40]. Given the size of the estimated associations and alignment with prior evidence, we believe it unlikely that differences between ABC and TFV backbones would be fully explained by “healthy user” bias. However, further study of incident NRTI users, using target trial methodology, for example, would strengthen insights. Finally, REPRIEVE did not collect reasons for changes to ART regimen, and there may be unmeasured confounding of effect estimates.

In conclusion, increased hazard of MACE was observed among ABC- compared to TAF- or TDF-containing regimens in this analysis of the REPRIEVE cohort. This effect persisted after weighting to limit confounding and selection biases and adjusting for cardiovascular risk factors including ASCVD risk and eGFR. This is the first analysis of a global randomized clinical trial of PWH with low-to-moderate cardiovascular risk taking contemporary ART with adjudicated cardiovascular endpoints to suggest ABC increases CVD hazard, corroborating several prior observational studies that included both high- and low-risk individuals. Since the REPRIEVE cohort features only PWH at low-to-moderate cardiovascular risk, our findings should be interpreted in the context of the study population. However, they also suggest that the CVD risk of ABC is not driven by known risk factors that may be more prevalent in high-risk individuals. With the availability of potent and well-tolerated 2- and 3-drug ART regimens for both ART-naive and -experienced patients, it is no longer necessary to include ABC in the ART armamentarium. Although more detailed investigations regarding the ASCVD impact of new and contemporary ART agents are needed, the results of our analysis indicate it would be most prudent to avoid ABC use in order to limit ASCVD risk in PWH.

## Notes

***Acknowledgments.*** The study investigators thank the study participants, site staff, and study-associated personnel for their ongoing participation in the trial. In addition, we thank the following: the AIDS Clinical Trials Group (ACTG) for clinical site support; ACTG Clinical Trials Specialists (Laura Moran, MPH, and Jhoanna Roa, MD) for protocol development and implementation support; the data management center, Frontier Science Foundation, for data support; the Center for Biostatistics in AIDS Research for statistical support; and the Community Advisory Board for input for the community.

***Author contributions.*** E. D. S., with the supervision of H. J. R. and assistance of M. W., developed the statistical analysis plan and performed the statistical analysis. C. M., C. J. F., J. A. A., M. R. D., S. K. G., and H. R. provided statistical review. E. D. S. and C. M. wrote the initial draft of the manuscript and revised subsequent drafts. C. M., C. J. F., J. A. A., M. R. D., S. K. G., P. S. D., R. B., J. S. C., and G. S. B. reviewed all versions of the manuscript and provided clinical review. All authors contributed to the interpretation of the data and reviewed the final draft of the article.

***Ethics statement.*** Each clinical research site obtained institutional review board/ethics committee approval and any other applicable regulatory entity approvals. Participants were provided with study information, including discussion of risks and benefits, and signed the approved declaration of informed consent.

***Financial support.*** This work was supported by the National Institutes of Health (NIH) (grant numbers U01HL123336 and 1UG3HL164285 to the Clinical Coordinating Center and U01HL123339 and 1U24HL164284 to the Data Coordinating Center); Kowa Pharmaceuticals America, Inc; Gilead Sciences; and ViiV Healthcare. In addition, the National Institute of Allergy and Infectious Diseases (NIAID) supported this study through grants UM1 AI068636, which supports the ACTG Leadership and Operations Center; UM1 AI106701, which supports the ACTG Laboratory Center; and T32 AI007358, which supports the HIV Training Grant (to E. D. S.). This work was also supported by the Nutrition Obesity Research Center at Harvard (grant number P30DK040561 to S. K. G.).
